# Investigating the Use of Electronic Well-being Diaries Completed Within a Psychoeducation Program for University Students: Longitudinal Text Analysis Study

**DOI:** 10.2196/25279

**Published:** 2021-04-22

**Authors:** Myles-Jay Anthony Linton, Sarah Jelbert, Judi Kidger, Richard Morris, Lucy Biddle, Bruce Hood

**Affiliations:** 1 Population Health Sciences Bristol Medical School University of Bristol Bristol United Kingdom; 2 School of Education University of Bristol Bristol United Kingdom; 3 School of Experimental Psychology Bristol Cognitive Development Centre University of Bristol Bristol United Kingdom

**Keywords:** psychoeducation, diary, students, text analysis, wellbeing, science of happiness, university, emotional tone, e-mental health, mobile phone

## Abstract

**Background:**

Psychoeducation has the potential to support students experiencing distress and help meet the demand for support; however, there is a need to understand how these programs are experienced. Web-based diaries are a useful activity for psychoeducation because of their therapeutic benefits, ability to capture naturalistic data relevant to well-being, and appropriateness for text analysis methods.

**Objective:**

This study aims to examine how university students use electronic diaries within a psychoeducation program designed to enhance mental well-being.

**Methods:**

The Science of Happiness course was administered to 154 undergraduate students in a university setting (the United Kingdom). Diaries were collected from the students for 9 weeks. Baseline well-being data were collected using the Short Warwick-Edinburgh Mental Wellbeing Scale (SWEMWBS). The percentage of negative and positive emotion words used in diaries (emotional tone) and use of words from five life domains (social, work, money, health, and leisure) were calculated using the Linguistic Inquiry and Word Count 2015 software. Random effects (generalized least squares) regression models were estimated to examine whether time, diary characteristics, demographics, and baseline well-being predict the emotional tone of diaries.

**Results:**

A total of 149 students participated in the diary study, producing 1124 individual diary entries. Compliance with the diary task peaked in week 1 (n=1041, 92.62%) and was at its lowest in week 3 (n=807, 71.81%). Compared with week 1, diaries were significantly more positive in their emotional tone during week 5 (mean difference 23.90, 95% CI 16.89-30.90) and week 6 (mean difference 26.62, 95% CI 19.35-33.88) when students were tasked with writing about gratitude and their strengths. Across weeks, moderate and high baseline SWEMWBS scores were associated with a higher percentage of positive emotion words used in diaries (increases compared with students scoring low in SWEMWBS were 5.03, 95% CI 0.08-9.98 and 7.48, 95% CI 1.84-13.12, respectively). At week 1, the diaries of students with the highest levels of baseline well-being (82.92, 95% CI 73.08-92.76) were more emotionally positive on average than the diaries of students with the lowest levels of baseline well-being (59.38, 95% CI 51.02-67.73). Diaries largely focused on the use of social words. The emotional tone of diary entries was positively related to the use of leisure (3.56, 95% CI 2.28-4.85) and social words (0.74, 95% CI 0.21-1.27), and inversely related to the use of health words (−1.96, 95% CI −3.70 to −0.22).

**Conclusions:**

We found evidence for short-term task-specific spikes in the emotional positivity of web-based diary entries and recommend future studies examine the possibility of long-term impacts on the writing and well-being of students. With student well-being strategies in mind, universities should value and encourage leisure and social activities.

## Introduction

### Background

There is mounting concern regarding increases in the number of young people (aged 16-24 years) reporting long-standing mental health difficulties [1]. Rising university attendance among school-leavers approaching the end of adolescence has been observed globally [2], making universities an increasingly advantageous setting for providing mental health and well-being interventions [3]. Alongside treatment-based approaches for managing mental ill-health, there is a need to better understand the use of more accessible preventative approaches that equip students with resources to protect and promote their well-being [4].

### Student Mental Health and Well-being

The mental health of university students has been a priority in the higher education sector for at least a century [5]. In the last decade, however, academics have described a growing crisis in university students’ mental health [6,7]. For many students, university education is undertaken at a crucial period of transition into adulthood, associated with social, psychological, and developmental challenges across multiple life domains [8,9]. The World Health Organization’s World Mental Health International College Student project in 8 countries found that 31% of students had experienced a mental health disorder (such as anxiety or depression) within the previous 12 months [10]. As demand from students for counseling support continues to exceed supply, there is growing pressure to invest in strategies with the potential to prevent students from reaching the point where high-intensity one-on-one support is required [11].

### Embedding Psychoeducation in University

Psychoeducation, which involves the provision of information, tips for self-management, and guidance for staying well [12], may be one method for embedding well-being–enhancing interventions within university life. In a framework adapted from World Health Organization guidelines, experts have highlighted that alongside formal specialized treatment for severe mental ill-health problems, there is a need for structured and unstructured support for students experiencing varying levels of distress [13]. In line with this model, psychoeducation interventions can be used to facilitate social support networks and provide space for students to engage in self-care strategies. Research into university-based psychoeducation continues to expand [14,15], and there is a need to understand how students, with varying levels of well-being, experience and engage with these interventions.

### Practical and Methodological Use of Diaries

Diaries are a promising tool for integration into psychoeducation programs because of their potential for both therapeutic benefit and data collection [16]. Diaries offer writers a naturalistic space to reflect on experiences, and provide researchers with a depth of detail that is difficult to accurately achieve when relying on tools that require participants to retrospectively recall events from their past [17].

One of the earliest diary studies examining well-being in university found that common worries included academic stress and common sources of happiness included friendships [18]. Previous research with students has also indicated that participation in writing tasks about positive life events may lead to improvements in mood [19]. In a study where students were asked to write about their thoughts and feelings in relation to starting university, students who screened positive for depression (Beck Depression Index scores above 14) used significantly more negative emotion words than students without depression (scores below 7) [20]. These studies highlight a link between the emotional content of writing and how students report feeling about their mental health and well-being. Less is known about the balance between negative and positive feelings [21] and the emotional tone (balance of positive and negative words) of writing [22].

### Technological Developments in Diary Methodology

The availability and progression of technology has been critical in the ongoing advancement of diary-based research. In the field of diary analysis, researchers have used technology to identify prospective participants on the web [23], automatically prompt participants with poor compliance to diary completion tasks [24], and automatically monitor the time of day when respondents choose to complete their diaries [25].

Significant advancement has also been made in the analysis of open-text data contained within diaries. Natural language processing methods are being increasingly used to automate the analysis of diaries, as seen in a web-based eating disorder intervention [26]. Statistical models have been developed to predict depression severity based on the use of emotional words within social media posts [27]. The use of technology to enhance aspects of the research process, however, should not be undertaken without careful consideration. For example, where tasks are automated, it remains crucial to understand how the process is undertaken and be mindful of any trade-offs involved in generating these efficiencies.

### This Study and Research Questions

This study aims to examine how university students use electronic diaries within a psychoeducation program designed to develop mental well-being skills (Science of Happiness course). Longitudinal diary data enabled us to examine how the program was experienced and how the proportion of positive and negative emotion words (emotional tone) within diaries fluctuated over time. Automated text analysis methods were used to analyze the written content of the diaries. This study has 5 key research questions:

1. How compliant are students to the web-based diary task across weeks?
2. How does the proportion of positive and negative emotion words (emotional tone) within the diaries develop over time?
3. How are time, sociodemographics, diary characteristics, and baseline self-reported well-being related to the emotional tone of diaries? (model 1)
4. Is the trajectory of emotional tone within diaries dependent on baseline levels of well-being? (model 2)
5. Which life domains do students discuss most in their diaries, and how do these topics relate to the emotional tone of diary entries? (model 3)

## Methods

### Participants

The sample consisted of 154 university undergraduate students at a university in the UK. The *Science of Happiness* course was offered to students on 14 undergraduate courses in their first year of study (including *study abroad programs*) in exchange for academic credits. The course began in the first semester of the study (September 2019) and involved weekly lectures, weekly group tasks, and weekly diary entries (the focus of this research). The main evaluation examining the impact of the intervention is presented in a publication submitted separately [28].

### Data Collection

Sociodemographic characteristics and well-being data were collected at baseline. Parallel to the intervention, linked anonymized diary entries were collected digitally on a weekly basis at 9 time points.

Each student was assigned a study participant number, and no personally identifiable data were included in the research data set. Diary data were extracted from the web platform they were submitted to (Blackboard) and deposited in a Microsoft Excel spreadsheet stored on a secure server. Within the software, students were only able to submit one diary entry per week. The study obtained ethical approval from the University of Bristol Faculty Ethics Review Committee (reference: 27061987862).

### Electronic Diaries

The diaries we collected were *solicited*, given that they were requested by us for this study, rather than being spontaneous [29]. Students were able to submit their diary entries on the web via a desktop or laptop or smartphone. Students were prompted to write in their diaries about events in the week that had influenced their well-being or they were given guidance to write about their well-being in relation to specific topics such as their goals for the future. These tasks are described in Multimedia Appendix 1. Although this imposed some structure on the task, care was taken to encourage open responses and space for respondents to reflect without excessive constraints [29].

### Measures

A brief sociodemographic survey was used to capture data on gender, age, nationality, and ethnicity. Mental well-being was measured using the 7-item Short Warwick-Edinburgh Mental Wellbeing Scale (SWEMWBS) [30]. The original full-length Warwick-Edinburgh Mental Wellbeing Scale has demonstrable content validity, structural validity, criterion validity, internal consistency, and test-retest reliability across a UK nationally representative sample of adults and a specific sample of students in 2 UK universities [31]. The shortened 7-item version of the scale has been found to be internally consistent [30], and data on UK national norms have since been published to aid in interpreting scores [32]. Responses to the SWEMWBS are scored on a 5-point scale ranging from 1 (none of the time) to 5 (all of the time). Total scores were transformed, and higher scores indicated greater levels of mental well-being. In the regression analysis, well-being was recoded into a 3-level variable representing scores in the first (low), second (moderate), and third (high) tertiles of scores.

### Text Analysis

This study used a *dictionary method* approach to text analysis. This deductive approach involved automating the analysis of diary content based on a predetermined dictionary-based coding scheme [33].

#### Data Processing and Cleaning

All diaries were completed in the English language. Text data were stored in Microsoft Excel in a wide format (one participant per row and 1 week of diary entries per column). Typos were managed using guidance developed by the Language Use and Social Interaction lab [34]. A manual rather than an automated process was selected to preserve as much of the text as possible in its original form.

All diaries were manually scanned for typos, and words such as *roominate* were changed to *ruminate* to ensure the text analysis software was able to correctly identify the words used. Words such as *kinda* were not corrected because of the ability of text analysis software to recognize and correctly classify *slang text-speech*. In total, 892 words were manually corrected during the data cleaning process.

#### Linguistic Inquiry and Word Count Text Analysis Variables

Linguistic Inquiry and Word Count (LIWC) 2015 is a stand-alone piece of software that has been empirically validated to analyze the linguistic, social, and psychological content of text data [35]. LIWC 2015 uses an internal dictionary of almost 6400 words coded into different categories and counts the frequency of words used within any target text data. The software is now in its fourth major revision [22]. In a sample of 117,779 pieces of text data (novels, tweets, natural speech, expressive writing, and Twitter), LIWC 2015 correctly classified an average of 85.18% of words (SD 5.36%) [22]. In this study, we generated 7 LIWC text variables: positive emotions (eg, love, nice, and sweet), negative emotions (eg, hurt, ugly, and nasty), social (eg, mate, talk, and they), work (eg, job, class, and boss), health (eg, clinic, flu, and pill), leisure (eg, cook, TV, and movie), and money (eg, audit, cash, and owe). Each variable reflects the total number of words within each diary entry that falls into these prespecified categories of the LIWC’s internal dictionary. We also used LIWC to automatically calculate the word count of each diary. Using these variables builds on the use of sentiment analysis approaches in dictionary-based text analysis [33].

#### Emotional Tone

We also used the emotional tone variable generated by the LIWC software. This reflects the ratio of positive and negative emotion words. If a diary entry contained 100 words and 5 of its words were found in the *negative emotion* section of LIWC’s internal dictionary and 5 of its words were found in the *positive emotion* section of LIWC’s internal dictionary, the *positive emotion* score would be 5, the *negative emotion* score would be 5, and the *emotional tone* score would be 50. Higher scores on the emotional tone variable indicate a greater ratio of positivity within the text of diary entries, with scores above 50 indicating a greater proportion of positive words and scores below 50, indicating a greater proportion of negative words.

### Statistical Analysis

Data were analyzed using STATA 16 (StataCorp LLC) [36]. Sociodemographic characteristics (age, gender, ethnicity, and nationality) and diary characteristics (word count, diary entries, and emotional tone) were summarized descriptively for the whole sample. Participants’ characteristics were compared descriptively according to levels of compliance to the diary task. Diaries were also subgrouped based on word count to compare the characteristics of students who wrote the most and least.

The trajectory of emotional tone across weeks is displayed in line graphs. The trajectory of emotional tone seen in the whole data set (available-case analysis) was compared with the trajectory seen for the subsample with full compliance to diary entry (complete-case analysis).

We applied 4 models using random effects generalized least squares with an autoregressive disturbances regression approach (Table 1). This method was selected because the diaries in our data set are clustered around repeated measures from the same individuals and are thus not independent and because of the need for a method that is robust to variations in the number of repeated measures collected across individuals. Model 1 addressed research question 3, model 2 addressed research question 4, and model 3 addressed research question 5. Model 0 (a basic model with only week effects) was only estimated to establish whether our main model (model 1) had an improved statistical model fit (Wald chi-square) following the addition of sociodemographics, diary characteristics, and baseline well-being variables.

**Table 1:** Random effects generalized least squares with autoregressive disturbances regression models estimated.

Model	Sample (n=154), n (%)	Diaries (n=1124), n (%)	Dependent variable	Independent variables
Model 0: investigating the role of week effects only on the emotional tone of diary entries	110 (71.43)^a^	855 (76.07)	Emotional tone	Time (weeks)
Model 1: investigating the role of week effects, sociodemographics, diary characteristics, and baseline well-being on the emotional tone of diary entries	110 (71.43)	855 (76.07)	Emotional tone	Time (weeks), gender, age, diary word count, total diary entries, and baseline well-being
Model 2: investigating interaction effects between baseline well-being and week (Multimedia Appendix 2)	110 (71.43)	855 (76.07)	Emotional tone	Time (weeks), gender, age, diary word count, total diary entries, baseline well-being, and interactions between baseline well-being and time
Model 3: investigating the relationship between word use in 5 life domains on the emotional tone of diary entries	120 (77.92)	927 (82.47)	Emotional tone	Time (weeks), social words, work words, health words, money words, leisure words, gender, age, diary word count, and total diary entries

^a^Sample of respondents with complete data on variables in model 1 to enable Wald chi-square model fit comparisons (Multimedia Appendix 3).

## Results

### Sociodemographic and Diary Characteristics

A summary of the sample characteristics is provided in Table 2. Of a total sample of 154 participants, 149 (96.8%) participated in the diary completion activity, resulting in a total of 1124 diary entries. Participants had a mean age of 19.29 years (SD 1.47 years) and were predominantly female (93/124, 75.0%). The sample mainly consisted of White (100/123, 81.3%) and UK nationality students (96/121, 79.3%). Participants, on average, completed a mean of 7.54 diary entries (SD 1.47), and diary entries had a mean word count of 209.63 (SD 165.79). The text analysis software LIWC correctly captured 92% of the 235,621 words analyzed in this study, which is in line with expectations from previous LIWC analyses. The mean emotional tone of diary entries was 75.15 (SD 28.60).

**Table 2:** Total sample sociodemographics.

Sample characteristics			Values
Age (years; n=124), mean (SD)			19.29 (1.47)
**Gender (n=124), n (%)**			
	Female	93 (75.0)	
	Male	29 (23.4)	
	Prefer not to say	2 (1.6)	
**Ethnicity (n=123), n (%)**			
	Asian or Asian British	12 (9.8)	
	Black, African, Caribbean, or Black British	3 (2.4)	
	Mixed or multiple ethnic groups	5 (4.1)	
	Other ethnic group	3 (2.4)	
	White	100 (81.3)	
**Nationality (n=121), n (%)**			
	British	96 (79.3)	
	All other nationalities	25 (20.7)	
**Diary characteristics (n=149), mean (SD)**			
	Word count	209.63 (165.79)	
	Diary entries	7.54 (1.47)	
	Emotional tone	75.15 (28.60)	

From 855 diaries, the SWEMWBS scores were recoded by tertiles for the diaries of students with low well-being (range 14.08-19.98; n=296), moderate well-being (range 20.73-22.35; n=345), and high well-being (range 23.21-25.03; n=214).

**Figure 1:**
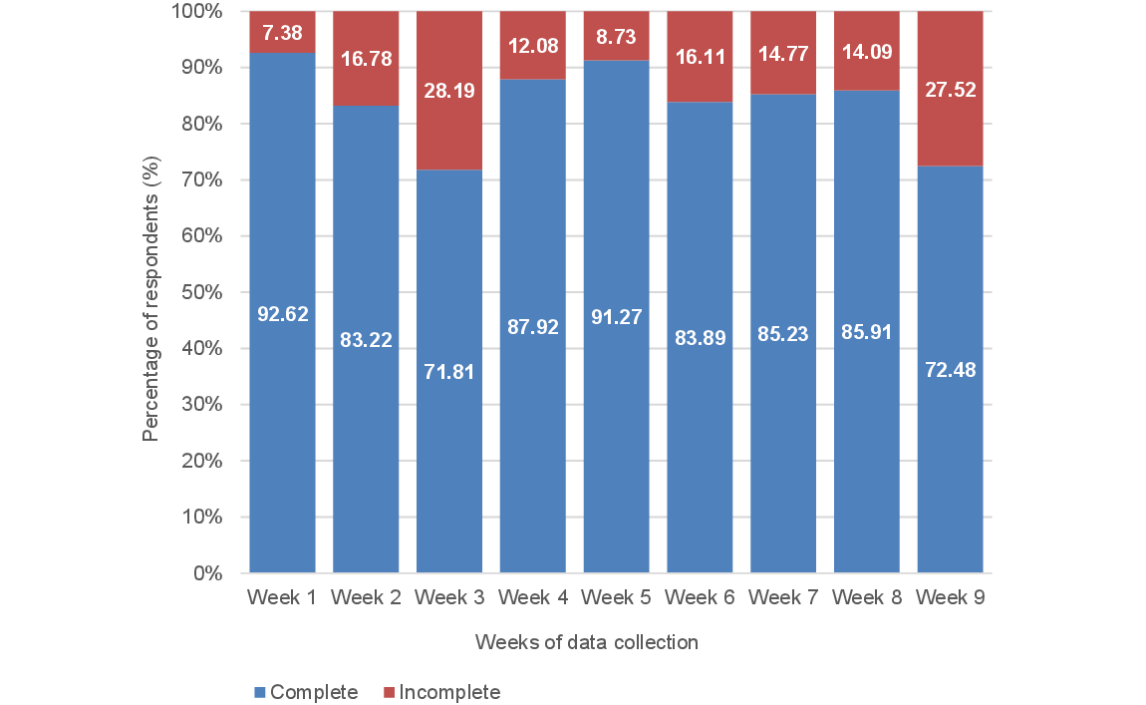
Compliance with diary completion activity.

Compliance with the diary task by week is displayed in Figure 1, which indicates that week 1 (138/149, 92.6%) and week 5 (136/149, 91.3%) had the highest compliance rates, whereas week 3 (107/149, 71.8%) and week 9 (108/149, 72.5%) had the lowest compliance rates.

### Diary Entry Compliance—Subgroup Analysis

Participants were grouped by those who completed all 9 entries (44/149, 29.5%), 8 entries (45/149, 30.2%), 7 entries (32/149, 21.5%), or fewer than 6 entries (28/149, 18.8%). Descriptive statistics for the characteristics of participants across the 4 levels of diary compliance are presented in Table 3. Participants who were fully compliant with the diary activity (9 entries) had higher diary word counts (mean 242.96, SD 201.88) than participants who completed 6 or fewer diary entries (mean 175.00, SD 160.59). Furthermore, participants who were fully compliant had the highest levels of baseline well-being (mean 22.16, SD 2.57) compared with students with 6 of fewer diary entries (mean 20.78, SD 2.69). The emotional tone and percentage of females was highest for participants in the most compliant group; however, the pattern across levels of compliance was less clear.

**Table 3:** Key sample and diary characteristics by diary-compliance subsamples.

Characteristics	Diary compliance			
	9 entries	8 entries	7 entries	≤6 entries
Diaries (n=149), n	396	360	224	396
Word count (n=149), mean (SD)	242.96 (201.88)	213.00 (142.24)	167.53 (112.14)	175.00 (160.59)
Age (years; n=124), mean (SD)	19.26 (1.38)	19.26 (1.54)	19.04 (0.74)	19.74 (2.10)
Gender, female (n=124), n (%)	35 (28.2)	25 (20.2)	20 (16.1)	13 (10.5)
Baseline well-being (n=119), mean (SD)	22.16 (2.57)	20.91 (2.45)	21.46 (3.33)	20.78 (2.69)
Emotional tone (n=149), mean (SD)	77.39 (26.27)	75.33 (28.95)	70.68 (30.88)	75.53 (29.67)

### Word Count—Subgroup Analysis

There were no clear patterns in the age of respondents or emotional tone of diary entries based on the 4 word count categories (Table 4). Longer diary entries had the highest proportion of female respondents (155/281, 55.2% for diaries of *0-103.5* word count length and 195/280, 69.6% for diaries of *265+* word count).

**Table 4:** Key sample and diary characteristics by diary-compliance subsamples.

Characteristics	Word count (percentile range)			
	265+ (75-100)	163-265 (50-75)	103.5-163 (25-50)	0-103.5 (0-25)
Diary entries, n (%)	280 (24.9)	281 (25.0)	282 (25.1)	281 (25.0)
Age (years), mean (SD)	19.56 (1.56)	19.09 (1.21)	19.07 (1.20)	19.36 (1.64)
Gender (female), n (%)	195 (69.6)	182 (64.8)	178 (63.1)	155 (55.2)
Emotional tone, mean (SD)	72.48 (26.97)	79.33 (26.91)	74.13 (28.50)	74.67 (31.47)

### Trajectory of Diary Emotional Tone

**Figure 2:**
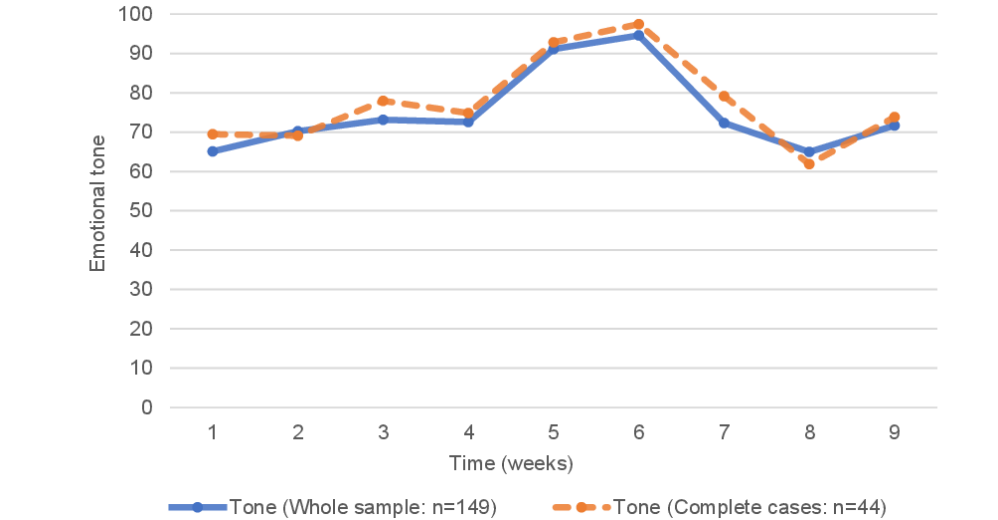
Trajectory of mean emotional tone within diaries for whole sample (and participants with complete data for all 9 weeks).

The pattern of scores for the available-case analysis and the complete-case analysis followed an inverted U shape (Figure 2), with a peak between weeks 5 and 6 when students are completing the gratitude and signature strengths diaries, respectively. Given the similarity in the trajectory pattern of emotional tone, whether complete case data or all available data were used, subsequent analyses in this paper are presented using the whole data set diary data (available-case analysis).

### Factors Determining the Emotional Tone of Diaries (Model 1)

The results from the random effects generalized least squares regression examining the role of week effects, sociodemographics, diary characteristics, and baseline levels of well-being in determining the emotional tone of diary entries are presented in Table 5. The emotional tone of diary entries in weeks 5 and 6 were, on average, higher by 23.90 (95% CI 16.89-30.90; *P*<.001) and 26.62 (95% CI 19.35-33.88; *P*<.001), respectively, in comparison with the emotional tone of diary entries in week 1. The mean emotional tone did not significantly differ from week 1 for the remaining 6 weeks. As the age of students increased by 1 year, the emotional tone of diary entries was higher, on average, by 1.98 (95% CI 0.51-3.46; *P*=.008). Students with both moderate (5.03, 95% CI 0.08-9.98; *P*=.046) and high (7.48, 95% CI 1.84-13.12; *P*=.009) levels of baseline well-being had diaries with significantly higher emotional tone compared with students with low baseline well-being. Gender and diary characteristics had no clear effects.

**Table 5:** Model 1: random effects generalized least squares regression examining the role of time (week effects), sociodemographics (age and gender), diary characteristics (word count and entries), and baseline well-being (moderate and high well-being, compared with low well-being) in determining the emotional tone of diary entries.^a^

Covariates					Coefficient			*P* value			95% CI
**Time**											
	Week 1			Reference^b^			N/A^c^			N/A	
	Week 2			4.57			.19			−2.24 to 11.38	
	Week 3			4.72			.20			−2.57 to 12.02	
	Week 4			3.78			.30			−3.33 to 10.89	
	Week 5			23.90^d^			<.001			16.89 to 30.90	
	Week 6			26.62^d^			<.001			19.35 to 33.88	
	Week 7			4.73			.19			−2.38 to 11.84	
	Week 8			−3.41			.35			−10.62 to 3.79	
	Week 9			4.56			.23			−2.91 to 12.03	
**Sociodemographics**											
	**Gender**										
		Male	Reference			N/A			N/A		
		Female	−2.44			.34			−7.48 to 2.59		
	Age			1.98^e^			.008			0.51 to 3.46	
**Diary characteristics**											
	Word count			−0.01			.10			−0.02 to 0.00	
	Total diary entries			0.43			.66			−1.47 to 2.33	
**Baseline well-being**											
	Low well-being			Reference			—			—	
	Moderate well-being			5.03^f^			.046			0.08 to 9.98	
	High well-being			7.48^e^			.009			1.84 to 13.12	

^a^Wald chi-square, *Χ*^2^_15_=137.0 (N=855); *P*<.001. This model provided an improved model fit, compared with model 0, which only included time (week effects) presented in Multimedia Appendix 3.

^b^Reference category for factor variables.

^c^N/A: not applicable.

^d^*P*<.001.

^e^*P*<.01.

^f^*P*<.05.

### Differences in Emotional Tone Across Weeks for Students With the Highest and Lowest Levels of Baseline Well-being (Model 2)

**Figure 3:**
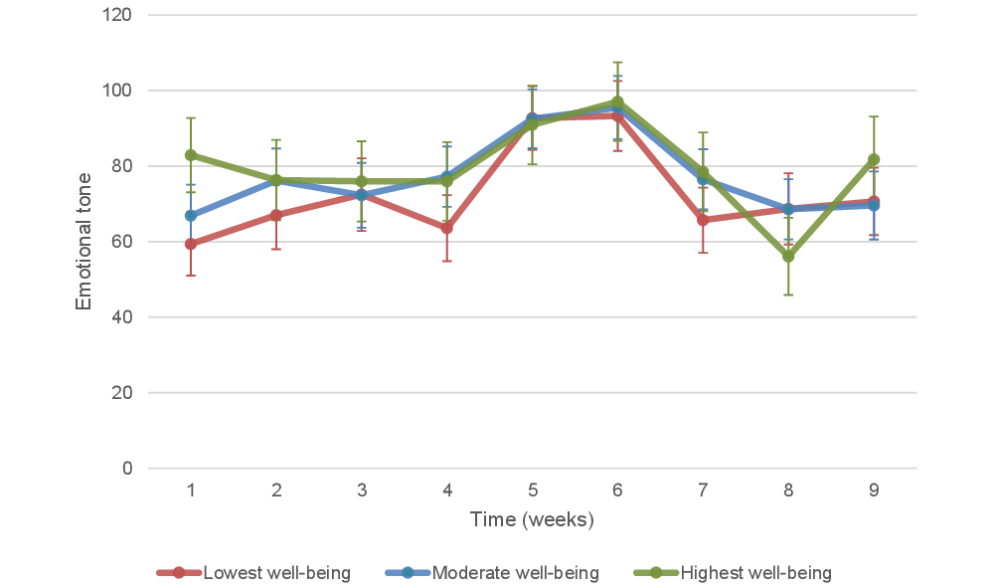
Trajectory of emotional tone for students with low, moderate, and high baseline well-being, adjusted for age, gender, word count, and total diary entries.

As a secondary analysis, we tested whether adding an interaction effect between baseline well-being (tertiles for the lowest, moderate, and highest scores) and time (weeks) improved the fit of the model (Multimedia Appendix 2). Adding this interaction to the model produced a significantly higher Wald chi-squared value (*P*=.03), indicating a better fitting model. To examine how the pattern of emotional tone differed between students with the lowest and highest well-being across the weeks, we plotted the mean emotional tone for students with the highest and lowest levels of well-being (adjusted for age, gender, word count, and total diary entries; Figure 3). The difference in the pattern was most substantial in the first week, where students with the lowest levels of baseline well-being started out with markedly lower average emotional tone of their diary entries. Furthermore, students with the highest levels of baseline well-being demonstrated a marked drop in the emotional tone of their diaries during week 8 when they were asked to write about their goals for the future.

### Life Domain Analysis (Social, Health, Leisure, Money, and Work; Model 3)

**Figure 4:**
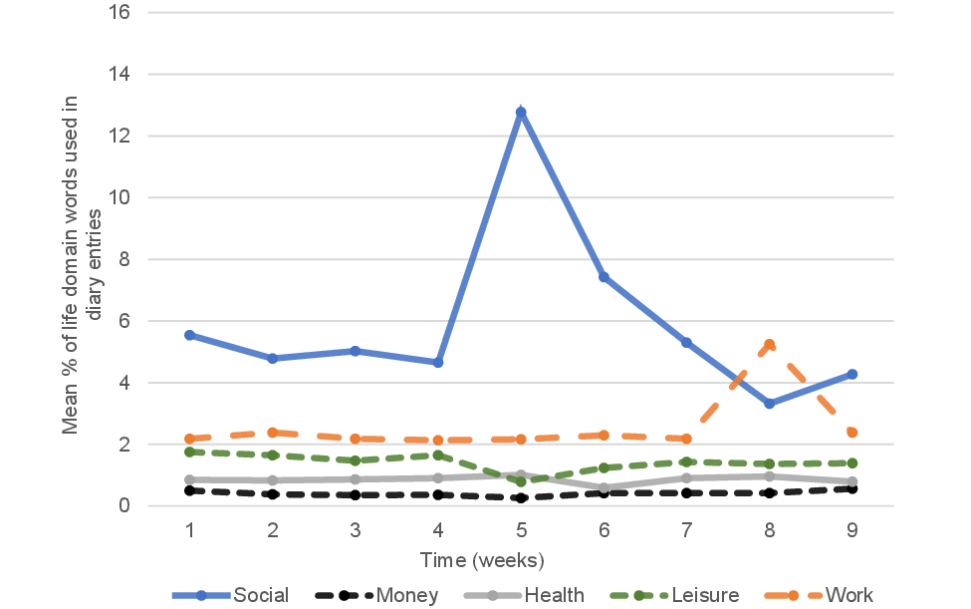
Use of life domain–specific terms in diary entries across weeks.

The use of words from 5 life domains (mean percentage of words per diary entry) across the 9 weeks is presented in Figure 4. Social topics were the most discussed topic apart from in week 8 (when participants were asked to diary about their goals) when work was the most dominant life domain discussed. A peak in the discussion of social topics was observed for week 5 (when participants were asked to diary about gratitude). Money was the least discussed topic of diaries for all 9 weeks.

Table 6 presents the results of the random effects generalized least squares regression examining how the use of words in the 5 life domains relates to the emotional tone of diary entries. As the percentage of *social* words in diaries increased by 1, the emotional tone of diaries increased by an average of 0.74 (95% CI 0.21-1.27; *P*=.006). As the percentage of *leisure* words in diaries increased by 1, the emotional tone of diaries increased on average by 3.56 (95% CI 2.28-4.85; *P*<.001). Finally, as the percentage of *health* words in diaries increased by 1, the emotional tone of diaries was more negative by an average of 1.96 (95% CI −3.70 to −0.22; *P*=.03). The relationships between *work-* and *money*-related words and the emotional tone of diary entries were nonsignificant.

**Table 6:** Model 3: random effects generalized least squares regression examining how the use of words from 5 life domains (social, health, leisure, work, and money) relates to the emotional tone of diary entries, controlling for week effects (time), sociodemographics (age and gender), and diary characteristics.^a^

Covariates				Coefficient		*P* value		95% CI
**Time**								
	Week 1		Reference^b^		N/A^c^		N/A	
	Week 2		5.61		.08		−0.70 to 11.91	
	Week 3		6.38		.07		−0.54 to 13.30	
	Week 4		5.69		.10		−1.05 to 12.44	
	Week 5		22.32^d^		<.001		14.51 to 30.13	
	Week 6		27.01^d^		<.001		20.08 to 33.93	
	Week 7		6.86		.05		0.13 to 13.59	
	Week 8		1.28		.73		−5.99 to 8.54	
	Week 9		6.45		.08		−0.66 to 13.57	
**Sociodemographics**								
	**Gender**							
		Male	Reference		N/A		N/A	
		Female	−4.68		.08		−9.91 to 0.54	
	Age		2.00^e^		.01		0.46 to 3.55	
**Diary characteristics**								
	Word count		−0.01		.13		−0.02 to 0.00	
	Total diary entries		0.32		.74		−1.58 to 2.22	
**Life domains**								
	Social		0.74^f^		.006		0.21 to 1.27	
	Health		−1.96^e^		.027		−3.70 to −0.22	
	Leisure		3.56^d^		<.001		2.28 to 4.85	
	Work		−0.16		.73		−1.03 to 0.71	
	Money		−0.80		.49		−3.07 to 1.46	

^a^Wald chi-square, *Χ*^2^_18_=189.7 (N=927); *P*<.001.

^b^Reference category for factor variables.

^c^N/A: not applicable.

^d^*P*<.001.

^e^*P*<.05.

^f^*P*<.01.

## Discussion

### Principal Findings

Compliance with the diary task peaked in week 1 (1041/1124, 92.62%) and the fewest diaries were completed in week 3 (807/1124, 71.81%). Students with the most completed diaries had the highest diary word counts, highest levels of baseline well-being, and on average had diaries with the highest emotional tone. Compared with week 1, diaries were significantly more positive in their emotional tone during weeks 5 and 6 when diary tasks involved writing about gratitude and strengths, respectively. This improvement in emotional tone was not observed at the end of the course, indicating a short-term rather than a lasting improvement in the emotional tone of writing. Higher levels of baseline well-being were associated with more emotionally positive diary entries, and the pattern of emotional tone within diaries across the weeks seen for students with low, moderate, and high baseline well-being was distinct. Diaries predominantly focused on social topics throughout the weeks, and the emotional tone of diaries was positively related to the use of leisure and social words and negatively related to the use of health words.

### Existing Literature

The dominant focus on social topics within well-being–oriented diaries reinforces findings from previous research, indicating that a lack of social connectedness predicted higher levels of related mental distress (anxiety and depression) in a cross-sectional study of UK university students [37]. In this study, students with the lowest levels of baseline well-being used on balance more negative words in their diaries (emotional tone), in line with findings linking experiences of depression with the use of negative words [20]. Our findings indicate a strong link between writing about leisure and the use of positive emotion words complements a recent study in China that reported when students were asked to draw their happiest moments, they often depicted leisure activities [38]. Despite links in the literature between financial circumstances and well-being among university students [39,40], our work indicated that in some student samples, money worries may not necessarily factor into the weekly well-being experiences of students.

### Implications

The analysis of word use across life domains highlights the importance of social factors and leisure in the lives of university students. As such, attempts to tackle student well-being concerns should continue to experiment with the utility of peer support networks, engaging familial support and responsible use of social media. In terms of the important role leisure plays in student well-being, universities are encouraged to ensure multiple options for leisure are readily available, and students are encouraged to select activities that are personally satisfying [41]. The promotion of leisure among students also has the potential to encourage participation in health-promoting behaviors, such as physical activity [42].

These findings also have implications for the ongoing development of the Science of Happiness course. We present evidence for students’ acceptance and willingness to engage in a reasonably rigorous schedule of weekly web-based written diary tasks. It is also noteworthy that one of the weeks with lower compliance to the diary entry task occurred when students were on a break from study (week 3), which provides us important insight into the level of engagement to expect when students are disengaged from university academic activities. This work also provides data-driven guidance as to which students may be more prone to disengage from the task (ie, males and students with lower levels of baseline well-being).

There are wider implications for how student data are used as technology develops. The text analysis methods described in this paper could readily be applied to routinely collected information from students, for example, in written requests for well-being support, to estimate levels of distress based on the use of emotional words. Any developments in this area should reflect on the ethical questions about privacy and student preferences raised by experts working in the area of learning analytics [43]. Students should be involved in these discussions, and work should be undertaken to determine the risks, benefits, and opportunities provided by increased analytic involvement in how students are supported.

### Limitations

This study has several limitations. Participants in this study were self-selected; therefore, the respondents in this study may not be representative of the wider student population. Available national data indicate that the UK university population is slightly more female and two-thirds of White ethnicity [44]; however, in our sample, both demographics were overrepresented. This means that the observed gender differences need to be interpreted with caution. Furthermore, the study may not have attracted students who did not believe they have any difficulties with their well-being. As the course was only offered to first-year undergraduate students, these results may not be generalizable to other undergraduate years, postgraduates, and PhD graduate students. Separately, as diary data were collected without the presence of researchers, we were unable to follow up with participants to explore any specific points raised in depth. Finally, without a control group also completing weekly diaries, in this study, we were unable to test whether specifically the diary task had a positive impact on the self-reported well-being of participants. However, on balance students demonstrated a high level of compliance with the diary task, the course generated novel data, and we were able to flexibly apply the LIWC software to analyze the content and underlying emotional tone of the available text data.

### Future Research

In this study of undergraduate students, diaries predominantly focused on social topics; however, future research could examine whether different patterns, such as a focus on money or health, are observed in nonstudent samples of young adults. This study could build on attempts to investigate how distinct the challenges experienced by students are to university populations [45,46]. Separately, it would be valuable to investigate whether money was more of a focus within the diaries of subgroups with different financial circumstances [47]. Although leisure was discussed less frequently than social topics, the use of leisure words was related to the emotional positivity of diary entries. Building on work that has theorized about the many dimensions of leisure in university settings [48], it would be valuable to conduct more in-depth research with students to examine how and when different forms of leisure contribute to their well-being.

Given the noticeable spike in emotional tone observed when students were tasked with writing diaries about gratitude, a future iteration of the course could focus solely on this subject [49]. Future research could examine the sources of gratitude for university students and explore whether repeated engagement in this task has a positive and sustained impact on the emotional positivity of writing and self-reported well-being. The more we understand about the relationship between what students write and how they feel, the better informed we will be to decide how far the use of these methods should be extended. We also recognize that a future analysis of unsolicited diaries would enrich our understanding of how diaries are used in nonexperimental settings.

One explanation for why compliance with the diary task fell to 72.5% (108/149) in week 9 is that this time point marked the conclusion of the course. Exploring this phenomenon and broader motivations for participation in the course would expand our understanding of how the course is interpreted and experienced. In future research, it would be informative to examine the acceptability of automated digital prompts designed to encourage the use of the web-based diaries, especially for participants who are willing yet have simply forgotten to complete the task.

### Conclusions

This study demonstrated the informative power of web-based diaries, the flexibility of computerized text analysis methods, and differential experiences of students with varying levels of baseline well-being engaged in psychoeducation. Students used their diaries with a high level of compliance and wrote with the highest proportion of positive emotion words during weeks where diaries focused on gratitude and strengths. Further research is needed to explore the importance of leisure to well-being the longer-term impact of diaries on well-being, and suggestions are provided for how the science of happiness could be adapted in the future. We present support for previous studies highlighting the importance of social factors and leisure for student well-being, and echo recommendations that universities should ensure these activities are facilitated and encouraged.

## Appendix

Multimedia Appendix 1 Diary tasks for each week of the study.

Multimedia Appendix 2 Model 2: random effects generalized least squares regression examining interaction effects between time and baseline well-being levels, controlling for sociodemographics and diary characteristics.

Multimedia Appendix 3 Model 0: random effects generalized least squares regression examining only week effects among samples with complete-case data in model 1.

## Abbreviations

LIWC: Linguistic Inquiry and Word Count
SWEMWBS: Short Warwick-Edinburgh Mental Wellbeing Scale
