# Supplementary material for: Investigating the Use of Electronic Well-being Diaries Completed Within a Psychoeducation Program for University Students: Longitudinal Text Analysis Study
Source: J Med Internet Res. 2021 Apr 22;23(4):e25279. doi: 10.2196/25279 (PMC8103302; doi:10.2196/25279)
Supplement: Multimedia Appendix 1 [file jmir_v23i4e25279_app1.docx]

**Multimedia Appendix 1 – Diary tasks for each week of the study**

Week 1: How did any events or experiences in the past week affect your levels of mental well-being? Please provide some description and insight.

Week 2: How did any events or experiences in the past week affect your levels of mental well-being? Please provide some description and insight.

Week 3: How did any events or experiences in the past week affect your levels of mental well-being? Please provide some description and insight.

Week 4: How did any events or experiences in the past week affect your levels of mental well-being? Please provide some description and insight.

Week 5: Gratitude: Write a letter of gratitude to someone who did something for you for which you are extremely grateful but to whom you never expressed your deep gratitude. Describe in specific terms what this person did, why you are grateful to this person, and how this person’s behaviour affected your life. Try to be as concrete as possible. Don’t worry about perfect grammar or spelling. Provide some description and insight.

Week 6: Signature strengths: How did any events or experiences in the past week, including the ‘Signature Strengths activity’, affect your levels of mental well-being? Please provide some description and insight. This activity involved students completing the Signature Strengths survey, undertaking an activity related that their signature strength, and reflecting on the experience in their diary.

Week 7: How did any events or experiences in the past week affect your levels of mental well-being? Please provide some description and insight.

Week 8: Goal setting: For this week's diary set yourself a goal using the Wish, Outcome, Obstacle, Plan (WOOP) goal-setting method. Use the diary to record your WOOP plan and or reflect on the experience.

Week 9: How did any events or experiences in the past week affect your levels of mental well-being? Please provide some description and insight.
