# Supplementary material for: Investigating the Use of Electronic Well-being Diaries Completed Within a Psychoeducation Program for University Students: Longitudinal Text Analysis Study
Source: J Med Internet Res. 2021 Apr 22;23(4):e25279. doi: 10.2196/25279 (PMC8103302; doi:10.2196/25279)
Supplement: Multimedia Appendix 2 [file jmir_v23i4e25279_app2.docx]

## Multimedia Appendix 2: Model 2^d^ - random effects generalized least squares regression examining interaction effects between Time and baseline wellbeing levels, controlling for socio-demographics and diary characteristics

| **Covariates** | | | **Coefficient** | **P value** | **95% CI** |
| --- | --- | --- | --- | --- | --- |
| **Time** | | |  |  |  |
|  | Week 1 | | Reference^e^ | — | — |
|  | Week 2 | | 7.64 | .18 | -3.63to 18.90 |
|  | Week 3 | | 13.09^c^ | .04 | 0.72 to 25.46 |
|  | Week 4 | | 4.21 | .48 | -7.60 to 16.03 |
|  | Week 5 | | 33.34^a^ | <.001 | 21.78 to 44.90 |
|  | Week 6 | | 33.91^a^ | <.001 | 21.65 to 46.17 |
|  | Week 7 | | 6.30 | .29 | -5.47 to 18.07 |
|  | Week 8 | | 9.31 | .14 | -3.08 to 21.70 |
|  | Week 9 | | 11.28 | .07 | -0.73 to 23.29 |
| **Socio-demographics** | | |  |  |  |
|  | **Gender** | |  |  |  |
|  |  | Male | Reference^e^ | — | — |
|  |  | Female | -2.73 | .30 | -7.48 to 2.59 |
| Age (years) | | | 1.98^c^ | .010 | 0.51 to 3.46 |
| **Diary characteristics** | | |  |  |  |
| Word count | | | -0.01 | .15 | -0.02 to 0.00 |
| Total diary entries | | | 0.41 | .68 | -1.47 to 2.33 |
| **Baseline wellbeing** | | |  |  |  |
|  | Low wellbeing | | Reference^e^ | — | — |
|  | Moderate wellbeing | | 7.52 | .21 | -4.16 to 19.20 |
|  | High wellbeing | | 23.55^a^ | <.001 | 10.63 to 36.46 |
| **Baseline wellbeing x time (interaction)** | | |  |  |  |
|  | Week 1 x Low wellbeing | | Reference^e^ | — | — |
|  | Week 2 x Moderate wellbeing | | 1.66 | .84 | -14.00 to 17.31 |
|  | Week 2 x High wellbeing | | -14.23 | .11 | -31.63 to 3.16 |
|  | Week 3 x Moderate wellbeing | | -7.72 | .37 | -24.67 to 9.23 |
|  | Week 3 x High wellbeing | | -20.04^c^ | .036 | -38.76 to -1.31 |
|  | Week 4 x Moderate wellbeing | | 6.11 | .46 | -10.16 to 22.37 |
|  | Week 4 x High wellbeing | | -11.19 | .23 | -29.51 to 7.13 |
|  | Week 5 x Moderate wellbeing | | -7.72 | .34 | -23.72 to 8.28 |
|  | Week 5 x High wellbeing | | -25.34^b^ | .006 | -43.53 to -7.16 |
|  | Week 6 x Moderate wellbeing | | -5.31 | .54 | -22.08 to 11.46 |
|  | Week 6 x High wellbeing | | -19.75^c^ | .037 | -38.35 to -1.14 |
|  | Week 7 x Moderate wellbeing | | 3.30 | .69 | -12.92 to 19.52 |
|  | Week 7 x High wellbeing | | -10.73 | .25 | -29.03 to 7.56 |
|  | Week 8 x Moderate wellbeing | | -7.64 | .37 | -24.32 to 9.04 |
|  | Week 8 x High wellbeing | | -36.14^a^ | <.001 | -54.72 to -17.56 |
|  | Week 9 x Moderate wellbeing | | -8.61 | .32 | -25.48 to 8.26 |
|  | Week 9 x High wellbeing | | -12.46 | .20 | -31.45 to 6.53 |

^a^*P*<.001.

^b^*P*<.01.

^c^*P*<.05.

^d^Wald chi-square, X^2^_31_=165.2 (N=855); *P*<.001.

^e^ Reference category for factor variables
