# Supplementary material for: Investigating the Use of Electronic Well-being Diaries Completed Within a Psychoeducation Program for University Students: Longitudinal Text Analysis Study
Source: J Med Internet Res. 2021 Apr 22;23(4):e25279. doi: 10.2196/25279 (PMC8103302; doi:10.2196/25279)
Supplement: Multimedia Appendix 3 [file jmir_v23i4e25279_app3.docx]

**Multimedia Appendix 3: Model 0^d^ - random effects generalized least squares regression examining only week-effects amongst sample with complete-case data in Model 1**

| **Covariates** | **Coefficient** | **P value** | **95% CI** |
| --- | --- | --- | --- |
| **Time** |  |  |  |
| Week 1 | Reference ^e^ | — | — |
| Week 2 | 5.01 | .15 | -1.77 to 11.79 |
| Week 3 | 4.97 | .18 | -2.30 to 12.24 |
| Week 4 | 4.37 | .22 | -2.68 to 11.42 |
| Week 5 | 24.09^a^ | <.001 | 17.12 to 31.06 |
| Week 6 | 27.22^a^ | <.001 | 20.01 to 34.43 |
| Week 7 | 5.25 | .14 | -1.80 to 12.29 |
| Week 8 | -2.63 | .47 | -9.76 to 4.50 |
| Week 9 | 5.18 | .17 | -2.18 to 12.53 |

^a^*P*<.001.

^b^*P*<.01.

^c^*P*<.05.

^d^Wald chi-square, X^2^_9_=118.5 (N=855); *P*<.001.

^e^ Reference category for factor variables
